# Immune microenvironment and clinical feature analyses based on a prognostic model in lymph node-positive breast cancer

**DOI:** 10.3389/fonc.2023.1029070

**Published:** 2023-03-22

**Authors:** Nannan Lu, Changfang Fu, Lei Zhang, Yangyang You, Xiang Li, Qian Zhang, Pin Wang, Xinghua Han

**Affiliations:** ^1^ Department of Oncology, The First Affiliated Hospital of University of Science and Technology of China (USTC), Division of Life Science and Medicine, University of Science and Technology of China, Hefei, Anhui, China; ^2^ Department of Pharmacy, The First Affiliated Hospital of University of Science and Technology of China, Division of Life Sciences and Medicine, University of Science and Technology of China, Hefei, Anhui, China; ^3^ Department of Cardiology, The First Affiliated Hospital of University of Science and Technology of China (USTC), Division of Life Sciences and Medicine, University of Science and Technology of China, Hefei, Anhui, China; ^4^ Chinese Academy of Sciences Key Laboratory of Soft Matter Chemistry, Department of Polymer Science and Engineering, University of Science and Technology of China, Hefei, Anhui, China; ^5^ Department of Oncology, Affiliated Anhui Provincial Hospital, Bengbu Medical College, Bengbu, Anhui, China; ^6^ Department of Gastroenterology, Affiliated Drum Tower Hospital, Medical School of Nanjing University, Nanjing, Jiangsu, China

**Keywords:** breast cancer, lymph node metastasis, tumor-infiltrating lymphocyte, LNPRS score, prognosis

## Abstract

**Background:**

If lymph node metastasis occurs in breast cancer patients, the disease can progress rapidly. Based on the infiltrative immune cells of breast cancer patients with lymph node positivity, we constructed the LNPRS for selecting prognostic predictors.

**Methods:**

The LNPRS was established and the predictive value of the LNPRS was verified by independent testing cohorts. A nomogram was also established to confirm the therapeutic guidance significance of the LNPRS. The correlation of the LNPRS with tumor mutation burden, immune microenvironment score, immune checkpoints, the proportion of tumor-infiltrating immune cells, and GSEA and GSVA enrichment pathways were also evaluated.

**Results:**

In the training cohort, the overall survival of breast cancer patients who had high LNPRS was shorter than that of patients who had low LNPRS (7.98 years versus 20.42 years, P-value< 8.16E-11). The AUC values for 5-, 10-, and 15-years were 0.787, 0.739, and 0.800, respectively. The ability to predict prognosis for the LNPRS was also tested in 3 independent testing cohorts. Furthermore, the predictive value of the LNPRS for chemotherapy and immunotherapy was also proven. The GSEA and GSVA showed that the LNPRS was closely related to the activation of T and B lymphocytes and IFN-γ secretion. Moreover, breast cancer patients with low LNPRS had higher TME scores than those with high LNPRS.

**Conclusion:**

We can conclude that the LNPRS is a robust prognostic biomarker in breast cancer patients with positive lymph nodes and may be helpful for patients to make a clinical decision.

## Introduction

According to a report from the CA-Cancer J Clin journal, breast cancer is the most important cause of death in women with tumors worldwide (1). New cases continue to slowly increase at a rate of 0.5% per year (1). According to data from the United States, the number of new cases of breast cancer will exceed 290,000 in 2022 (1). Currently, integrated treatment modes for breast cancer, including surgery, chemotherapy, hormonal therapy, targeted therapy, and radiation therapy, are used for patients who are diagnosed with breast cancer. However, there are still some patients, especially those with lymph node positivity, who progress rapidly after treatment.

Some research has shown that breast cancer is a highly immunogenic tumor, and the tumor immune microenvironment (TIME) is closely related to the development of breast cancer (2). Many immune cell types in the TIME significantly affect the prognosis of patients with breast cancer (2–4). Further research has found that tumor-infiltrating lymphocytes (TILs) are sensitive prognostic indicators for breast cancer (5–7). In addition, some studies have confirmed that TILs can predict the efficacy of chemotherapy and immunotherapy (8, 9).

Comprehensive and in-depth analysis of the characteristics of the breast cancer immune microenvironment may offer more sensitive and novel prognostic and therapeutic targets for patients. However, due to the limitations of the analysis methods and sample size, the prognostic and predictive value of TILs needs to be further improved. Wang et al. provided comprehensive immunogenomic analyses for the TIME of breast cancer patients and built a prognostic model by computational methods (10). However, the comprehensive analysis of the TIME of breast cancer with lymph node metastases is still limited. The characteristics analysis of infiltrative immune cells and identification of prognostic and predictive indicators for breast cancer patients with lymph node metastases still need to be addressed.

Therefore, we analyzed and quantified the immune microenvironment characteristics of breast cancer patients with positive lymph nodes by WGCNA based on the breast cancer training samples. Then, the least absolute shrinkage and selection operator (LASSO) Cox model was performed, and the lymph node positive related risk score (LNPRS) for breast cancer was constructed based on the training cohort samples (11, 12). Furthermore, the prognostic and predictive value of this prognostic model for survival time and therapeutic efficacy in the testing cohorts was investigated. Finally, the correlation of LNPRS with tumor mutation burden, distribution of 22 types of immune cells, and immune checkpoint molecules was explored and estimated.

## Materials and methods

### Collating and analyzing publicly available cohort datasets

The messenger RNA (mRNA) expression profiles for breast cancer cohorts were obtained from The Cancer Genome Atlas (TCGA). Five Gene Expression Omnibus (GEO) datasets for breast cancer cohorts with lymph node positivity and available overall survival time were collected from the public GEO database. The breast cancer cohorts with lymph node positivity and available overall survival time were also downloaded from the Molecular Taxonomy of Breast Cancer International Consortium (METABRIC) (13) and ArrayExpress (14). Finally, 2274 breast cancer patients from 9 independent cohorts in the study were selected.

We converted the probes into gene symbols by platform annotation file. The batch effects from different cohorts were removed (15). We normalized the raw count data and transformed the count data into transcripts per kilobase million (TPM) values.

The 482 breast cancer patients in the TCGA cohort were used to build the LNPRS as a training cohort. The 1112 breast cancer patients were analyzed from 3 independent cohorts of GSE20685, GSE97324, and METABRIC as testing cohorts. The overall survival and relapse-free survival in the GSE20685, GSE97324, and METABRIC cohorts were computed. The 680 breast cancer patients from the GSE130788, GSE140494, GSE18728, IMvigor210 and GSE78220 cohorts were used to evaluate the value of LNPRS in the response to chemotherapy and immunotherapy.

### TIME characteristics of breast cancer with lymph node positivity

The infiltration of 22 types of immune cells in the breast cancer patients was calculated, and the immune cell distribution between tumor and normal tissues is shown. The correlation among different immune cells in tumor samples was analyzed.

### Construction of the immune-related LNPRS model

Co-expression analysis of mRNA expression data and immune cell infiltration for breast cancer patients was performed, and the module-trait relationship map was plotted by weighted correlation network analysis (WGCNA) (16). TILs are closely related to antitumor immune response, including CD8^+^ T cells, activated CD4^+^ memory T cells, regulatory T cells (Tregs), and M1 macrophages (17). Thereafter, the network modules closely correlated with antitumor immune response were identified, and the magenta module was selected as an interesting module by referring to the correlation coefficient of modules. The expression profiles of module genes in the training and testing cohorts were obtained. The data on gene expression and patient survival for the training and testing cohorts were combined.

Then, six genes were identified from the module genes by univariable Cox proportional hazards regression analysis (P-value< 0.05). The P-values, hazard ratios (HRs), and 95% confidence intervals (CIs) of six prognosis-related genes were calculated, and a forest plot was drawn. The prognostic genes among the six genes were identified by LASSO Cox regression analysis and the LNPRS was established based on five genes (11, 12, 18). The correlation coefficients of these genes were computed (**Supplementary Table S1**). The LNPRS was constructed according to the following formula:


$$LNPRS\;score=\sum^{}\left(correlation\;coefficients\;for\;gene\;x\right)*\left(normalized\;gene\;x\;expression\;level\right)$$

### Validation of the LNPRS in multiple cohorts

The breast cancer patients from TCGA and GSE20685 were used as training and testing cohorts separately, and the samples were divided into high and low-risk groups based on the LNPRS by the optimal cutoff. The overall survival of patients in the training and testing cohorts was plotted based on high and low LNPRS. In different types, the Kaplan-Meier curve of individual genes was also present.

Next, the corresponding clinical information of breast cancer patients was also obtained from the public TCGA database, and the clinical variables of patients in the training cohort included age, sex, stage, and survival time. The alignment diagram, calibration curve of the nomogram diagram, risk curve, heatmap based on the gene signature, and ROC curve were plotted, and the results were shown. Independent prognostic analysis combining clinical features and LNPRS was performed by univariate and multivariate Cox regression analysis.

Then, the prognostic value of the LNPRS was verified by the samples of the GSE97324 and METABRIC cohorts. In the METABRIC cohort, 911 patients were divided into two groups with different levels of the LNPRS, and the LNPRS for every sample was obtained by the formula of the LNPRS scoring system. We plotted the curves of overall survival, relapse-free survival, and the ROC in the two groups. The correlation between the expression profiles of individual genes and survival time was examined. The relationships between the LNPRS value, tumor mutation burden (TMB), and survival time in the METABRIC cohort were also studied through the Wilcoxon test and Spearman test. In the GSE97324 cohort, the relationship between TNM stages and LNPRS values was compared by t-test, and Kaplan-Meier curves and ROC curves were also plotted.

### Correlation of tumor mutation burden, infiltrative immune cells, and LNPRS

The simple nucleotide variation data with breast cancer patients were searched. We downloaded masked somatic mutation data from the TCGA database. Gene mutation type and TMB data for every sample were obtained. We prepared the mutation annotation format (MAF) of somatic variants, and waterfall plots with mutation information of genes in each sample were shown by the visualization process (19). Differences and correlations of TMB in breast cancer patients with different risk levels based on the LNPRS were analyzed. The correlation between survival time, TMB, and LNPRS was assessed.

Next, we investigated the distribution of 22 types of immune cells (20). The correlation between the LNPRS and the proportion of immune cells in breast cancer patients was also analyzed. We evaluated the correlation between each independent prognostic gene based on the LNPRS scoring system and infiltrative immune cells by five immune cell analysis tools including XCELL, TIMER, QUANTISEQ, MCPCOUNTER, EPIC, CIBERSORT, and CIBERSORT-abs (21). The estimate score, stromal score, and immune scores in the training cohort were calculated with default parameters (22). Finally, TME scores with low or high LNPRS were evaluated and visualized.

### GSEA and GSVA analysis based on the LNPRS

The KEGG and GO pathway enrichment analyses of genes based on the LNPRS were performed by Gene Set Enrichment Analysis (GSEA) and Gene Set Variation Analysis (GSVA) for the training cohort (23, 24). The correlation between KEGG enrichment pathways and the LNPRS was analyzed and illustrated. We estimated the KEGG and GO enrichment pathways based on gene expression data.

### Predictive efficacy of the LNPRS in chemotherapy and immunotherapy

The drug sensitivity of the breast cancer patients from the TCGA cohort was analyzed. The mRNA expression data of genes associated with immune checkpoints in the training cohort were obtained. The immunophenoscore (IPS) of breast cancer patients from the TCGA database was downloaded from The Cancer Immunome Atlas (TCIA) (25). The correlation between 45 immune checkpoints and the LNPRS was calculated, and the forest plot was plotted. The sensitivity to PD-1 and CTLA4 antibodies of patients in the two groups with high and low LNPRS was studied.

We collected data from the GSE130788, GSE140494, and GSE18728 cohorts, and the three breast cancer cohorts contained complete transcriptome data and detailed clinical information for chemotherapy. We also obtained information on advanced urothelial cancer samples from the IMvigor210 cohort (26). Given the evaluation cycle and benefit time of immunotherapy, we deleted the survival data of patients with a survival time of fewer than 120 days in the IMvigor210 cohort. The GSE78220 datasets for the cohorts of metastatic melanoma samples were also downloaded from GEO data (27). We evaluated the predictive effect of the LNPRS in immunotherapy by the above two datasets.

The above processing were performed by glmnet, sva, DEseq2, limma, survival, survminer, reshape2 and ggplot2, ggpubr, GSEABase, GSVA, pRRophetic, org.hs.eg.db, DOSE, clusterProfiler, enrichplot, scales, ggtext, and tidyverse, pheatmap, timeROC, regplot, rms, complexheatmap, maftools, e1071, preprocessCore, corrplot, IMvigor210CoreBiologies, WGCNA, and estimate R packages.

### Statistical analysis

The samples in the different cohorts were divided into two groups according to the LNPRS. We plotted the Kaplan-Meier survival curves in the different cohorts. In the different groups, we estimated the differences in survival time, disease stage, and chemotherapy response by the two-sided log-rank test, t-test, or ANOVA test. We identified the gene signatures that were significantly correlated with overall survival time by univariate Cox regression analysis. We also assessed the independent prognostic value of the LNPRS by multivariable Cox regression analysis. The P-values, HR, and 95% CI for breast cancer cohorts were computed, and the risk prediction and clinical feature scores were shown visually by nomogram, which was calibrated and evaluated by a calibration plot (28).

All statistical analyses in the study were performed using R software (version 4.1.3), and the resulting P-values were two-sided. The P-values were considered statistically significant when they were less than 0.05.

## Results

### Analysis and qualification for immune microenvironment characteristics of breast tumor samples with lymph node positivity by WGCNA

**Figure 1:**
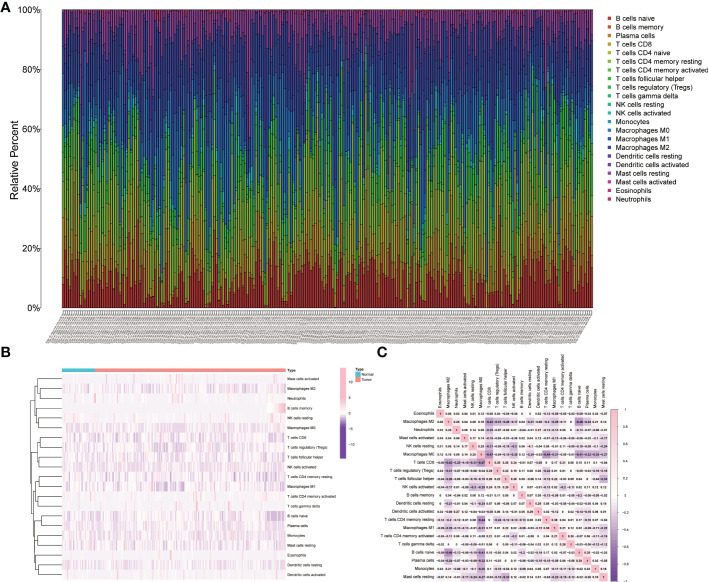
Relative analysis of immune cells in tumor samples and normal tissue. **(A)** The relative proportions of immune cells in the TCGA cohort. **(B)** The heatmap of the immune cells in the tumor samples and normal tissue. **(C)** The correlation analysis of infiltrative immune cells for the TCGA cohort.

The distributions of 22 types of infiltrating immune cells for the TCGA cohort are depicted, and the proportions of different immune cells in the normal or tumor samples are also illustrated in the heatmap (**Figures 1A, B**). The correlations of immune cells were also shown by the heatmap (**Figure 1C**).

**Figure 2:**
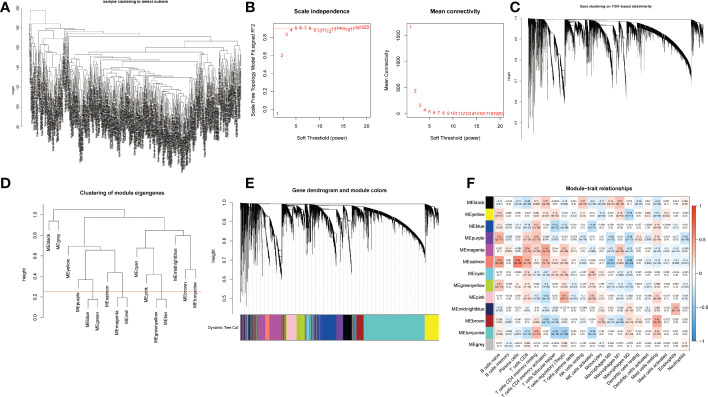
WGCNA of differentially expressed genes. **(A)** Sample clustering for detecting outliers. **(B)** Scale-free topology fitting index at different threshold values and mean connectivities. **(C)** Gene clustering on TOM-based dissimilarity. **(D)** Clustering of module eigengenes. **(E)** Gene dendrogram and module colors; **(F)** Module-trait relationships. Thirteen rows correspond to distinct co-expression modules, and twenty-one columns correspond to 21 types of infiltrative immune cells. The corresponding correlation coefficient and p-value for each type are shown in the diagram.

Differentially expressed genes (DEGs) and immune cells for each breast cancer sample in the training cohort were used for WGCNA (**Figure 2**). The outliers were detected and removed by a sample clustering dendrogram (**Figure 2A**). The optimal soft threshold power is selected by the scale-free topology model and the mean connectivity (**Figure 2B**). We have plotted the gene cluster dendrogram. In **Figure 2C**, the leaf represents a gene, and the branch on the tree represents a co-expression module. **Figure 2D** shows the clustering of module eigengenes. **Figure 2E** shows the clustering dendrograms for DEGs with dissimilarity. We calculated the correlations between each module and trait, and the correlation coefficients of the 13 modules and traits are shown (**Figure 2F**). The magenta module was chosen and analyzed by referring to the correlation coefficient and p-value.

### Establishment and evaluation of LNPRS in the breast cancer cohorts

**Figure 3:**
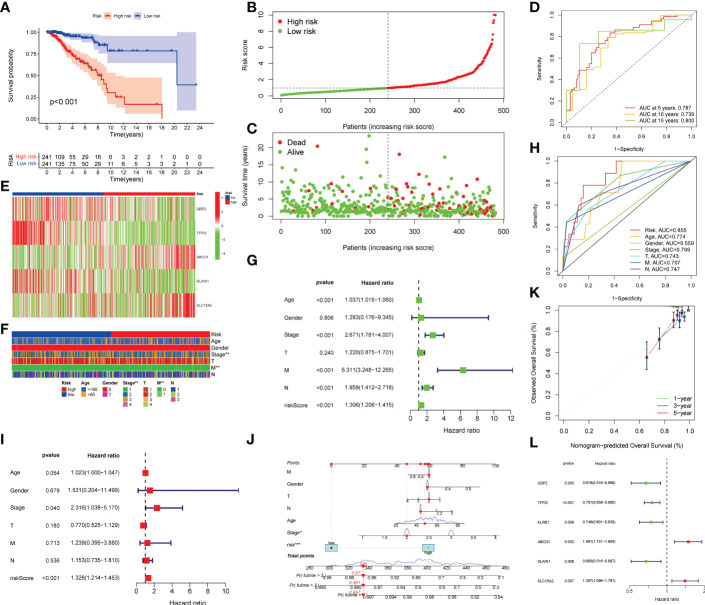
Prognostic value validation of the LNPRS in the training cohort. **(A)** The survival curve of patients in the high and low LNPRS groups. **(B, C)** The relation between the LNPRS and survival time of the patients with breast cancer. **(D)** The ROC curves of the LNPRS model for the TCGA cohort. **(E)** The heatmap of gene expression in different LNPRS groups. **(F)** The heatmap of patient clinical features in high and low LNPRS groups. **(G–I)** The P-values, HR, and 95% confidence interval of clinical features and riskScore by univariate **(G)** and multivariate **(I)** Cox regression analysis. **(H)** The AUC of clinical features and riskScore. **(L)** The P-value and HR of prognostic-related genes. **(J)** Nomogram diagram based on the LNPRS model. **(K)** The calibration plot of the nomogram.

**Figure 4:**
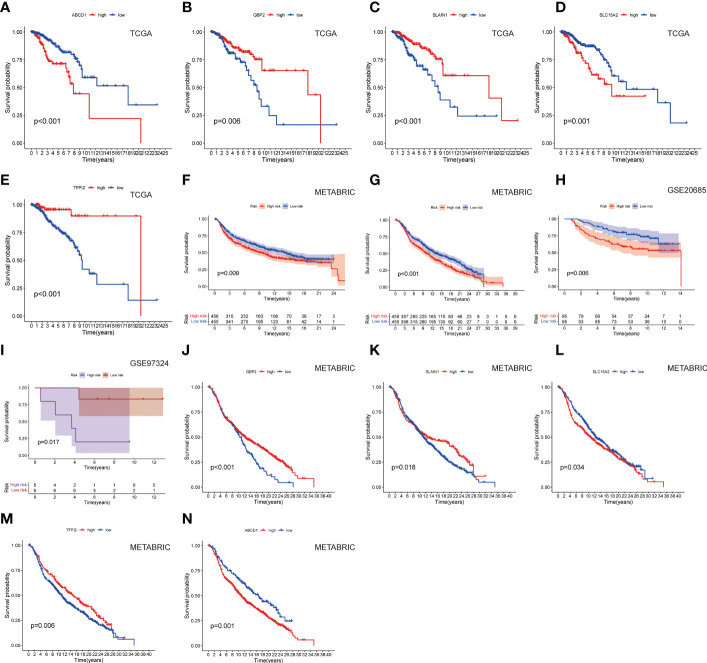
The survival curves of **(A)** ABCD1, **(B)** GBP2, **(C)** SLAIN1, **(D)** SLC15A2, and **(E)** TFPI2 based on LNPRS in the training cohort. The survival curves of the **(F)** METABRIC cohort (overall survival), **(G)** METABRIC cohort (relapse-free survival), **(H)** GSE20685 cohort, and **(I)** GSE97324 cohort. The survival curves of **(J)** GBP2, **(K)** SLAIN1, **(L)** SLC15A2, **(M)** TFPI2, and **(N)** ABCD1 in the METABRIC cohort.

The methods of LNPRS model building are referred to in the methods section of the article. Partial likelihood deviance and LASSO coefficient profiles are shown by vertical dotted line plots, and six genes were selected (**Figure 3L** **;** **Figures S1A, B**). LNPRS were established and included ABCD1, GBP2, SLAIN1, SLC15A, and TFPI2. As illustrated in **Figure 3A**, the breast cancer patients with high LNPRS in the training cohort had significantly shorter overall survival than those with low LNPRS (7.98 years versus 20.42 years, P-value< 8.16E-11; log-rank test; **Figure 3A**). The AUC values for 5, 10, and 15 years were 0.787, 0.739, and 0.800, respectively (**Figure 3D**). The correlation between survival time and the LNPRS for patients is indicated in **Figures 3B, C**. As the LNPRS increased, the death risk for patients gradually increased (**Figures 3B, C**). **Figure 3E** shows the heatmap of the correlation between death risk for patients and gene expression. The heatmap suggested that ABCD1 and SLC15A were high-risk genes, and GBP2, SLAIN1, and TFPI2 were low-risk genes (**Figure 3E**). The above results were the same as the findings of the survival curve of the single gene in the training cohort and METABRIC cohort (**Figures 4A–E** **;** **4J–N**).

**Figure 5:**
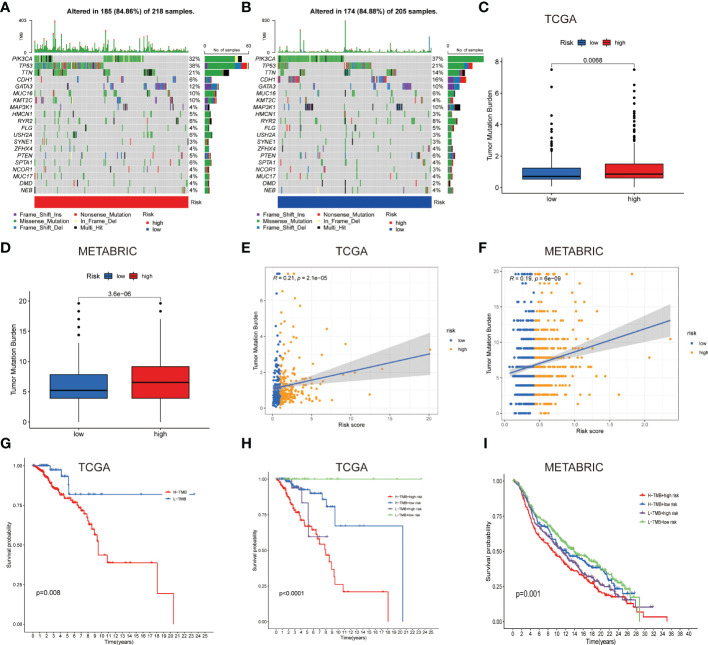
Gene mutation types and tumor mutation burden in the different cohorts. **(A, B)** Waterfall plots with mutation information of genes in the high **(A)** and low LNPRS groups **(B)**. **(C, D)** Boxplots of the differences in the tumor mutation burden with high or low-risk groups in the TCGA **(C)** and METABRIC cohorts **(D)**. **(E, F)** The correlation between tumor mutation burden and the LNPRS in the TCGA **(E)** and METABRIC cohorts **(F)**. **(G–I)** The survival curves for patients with high or low tumor mutation burden and LNPRS in the TCGA **(G, H)** and METABRIC cohorts **(I)**.

To further test the robustness of the LNPRS, the ability of the LNPRS to predict prognosis was also tested in the testing cohort (GSE20685), GSE97324, and METABRIC cohorts. With the same formula, we divided the patients into high and low LNPRS groups by optimal cutoff values. Similar results were observed in the testing cohort and other cohorts (**Figures 4F–I**). The performance of the LNPRS in the overall survival for the METABRIC cohort was assessed (10.16 years versus 13.05 years, P-value = 4.17E-4; log-rank test; **Figure 4G**). The relapse-free survival curve for the METABRIC cohort was represented graphically (9.17 years versus 13.98 years, P-value = 9.0E-3; log-rank test; **Figure 4F**). The AUC of the survival curve in the METABRIC cohort was computed (**Figure S2**). The patients in the GSE20685 cohort who had high LNPRS showed significantly shorter overall survival times than those who had low LNPRS (**Figure 4H**). The P-values of the GSE20685 and GSE97324 cohorts were 6.0E-3 and 1.7E-2, respectively, according to the log-rank test, (**Figures 4H, I**). The risk curve, time-dependent ROC curve, and heatmap of gene expression for the GSE20685 cohort are also shown in **Figures S2A–D**. The predicted performance of the single gene in the METABRIC cohort was consistent with the results of the TCGA cohort (**Figures 4J–N**). The ROC curves of the LNPRS for the IMvigor210, METABRIC, and GSE97324 cohorts are presented graphically in **Figure S4B, C**, **S5D**. The distribution of clinical features of the GSE97324 cohort in the high and low LNPRS groups is shown in **Figures S5A–C**. The correlation of the LNPRS with T stage and stage was evaluated, and the P-values in the GSE97324 cohort were 1.5E-2 and 4.2E-2, respectively (t-test; **Figures 5E, F**).

The prognostic value of clinical features and LNPRS in the TCGA cohort was further studied by univariate analysis. The heatmap for clinical features is presented in **Figure 3F**. As shown in **Figure 3G**, age, stage, M stage, N stage, and LNPRS were independent prognostic factors with a P-value< 0.001 in univariate regression analysis (**Figure 3G**). We conducted a multivariate regression analysis and found that the prognostic value of the LNPRS was independent of other clinical factors (**Figure 3I**). The LNPRS (P-value< 0.001) and stage (P-value = 0.040) were also independent prognostic factors in multivariate regression analysis (**Figure 3I**). In the training cohort, the AUC values of the LNPRS and clinical features were computed, and the LNPRS model obtained the highest value of 0.855 (**Figure 3H**). In the bar plot, the composition ratio of T, N, and M stages between the high and low LNPRS groups in the training cohort is illustrated (**Figures S3A–D**).

To provide a convenient and practical approach to predicting the prognosis of patients for clinical oncologists, a nomogram that incorporated the LNPRS and disease characteristics was constructed based on the breast cancer patients of the training cohort (**Figure 3J**). Based on the established nomogram, each patient obtained a score that could predict the overall survival time at 1-, 3- and 5-year (**Figure 3J**). When compared with the tumor features, the LNPRS contributed the most risk points (**Figure 3J**). The calibration curves of the training cohort are plotted in **Figure 3K**. The calibration curves and the ideal curve were very close, especially for the calibration curves of 3-year overall survival (**Figure 3K**). Therefore, these results indicated that the nomogram was clinically instructive and had good predictive performance.

### The difference in the tumor mutation burden between the patients with high or low LNPRS

To further study the differences in TMB, the TMB values of patients with different LNPRS were further analyzed. The waterfall plot in **Figures 5A, B** revealed the differences in the genetic mutations between the high and low LNPRS groups in the training cohort. Mutations in the TP53, PIK3CA, and TTN genes were more frequent, and the sample size and proportion of gene mutations in the two groups were similar (84.86% vs 84.88%; **Figures 5A, B**). The difference in TMB in the two risk groups was compared in the training cohort and METABRIC cohort and the patients with high LNPRS possessed higher TMB than those with low LNPRS by the Wilcoxon test. The P values were 6.8E-3 and 3.6E-6 in the TCGA and METABRIC cohorts, respectively (**Figures 5C, D**). The positive correlation between TMB and riskScore in the TCGA and METABRIC cohorts is illustrated in **Figures 5E, F** (R = 0.21, P-value = 2.1E-5 (TCGA cohort); R = 0.19, P-value = 6.0E-9 (METABRIC cohort); Spearman test; **Figures 5E, F**). In the 3 independent cohorts, the patients who possessed a low TMB and/or low LNPRS had longer overall survival times than those who possessed a high TMB and/or high LNPRS ((P-value = 8.0E-3, **Figure 5G**); (P-value< 1.0E-7, **Figure 5H**); (P-value = 3.6E-2, **Figure S4A**); (P-value = 1.0E-3, **Figure 5I**); log-rank test).

**Figure 6:**
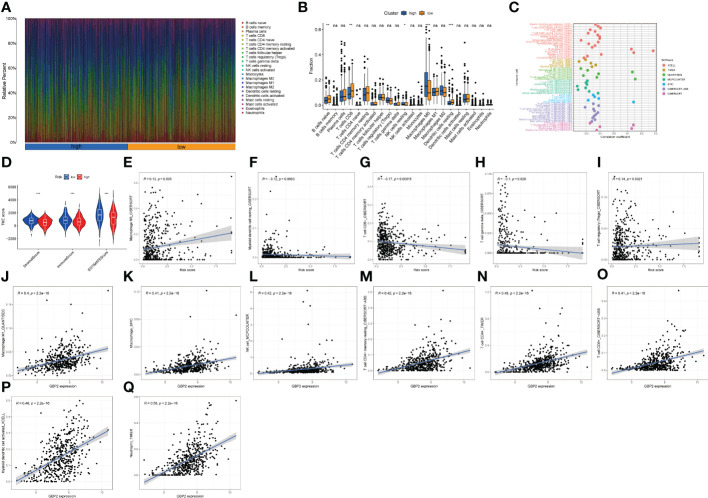
The proportion of immune cells, TME score, and infiltrative immune cells based on the LNPRS for the TCGA cohort. **(A, B)** The proportion of immune cells of patients with different LNPRS (ns ≥ 0.05, *< 0.05, **< 0.01, ***< 0.001). **(C)** The correlation analysis of the immune cells with LNPRS by 7 calculation methods. **(D)** The variance analysis of stromal score, immune score, and ESTIMATE score in the different risk groups. **(E–Q)** The correlation analysis of different immune cells and GBP2 gene expression.

Then, we studied the relationship between the LNPRS and immune microenvironment features. **Figure 6D** illustrates the relationship between the LNPRS and TME score, which included the stromal score, immune score, and ESTIMATE score, in the TCGA cohort. Moreover, breast cancer patients with low LNPRS had higher TME scores than those with high LNPRS (**Figure 6D**). There were significant differences between the two risk groups in stromal score, immune score, and ESTIMATE score, and the P-values were 1.60E-4, 4.20E-4, and 6.70E-5, respectively, according to the Wilcoxon test (**Figure 6D**).

### Immune cell proportion analyses based on LNPRS

The proportion of infiltrating immune cells for breast cancer patients with positive lymph nodes was observed and investigated, and the correlation between immune cells and LNPRS, and the proportions of infiltrating immune cells in the training cohort between different risk groups were studied by using 7 algorithms (TIMER, CIBERSORT, EPIC, XCELL, QUANTISEQ, MCPCOUNTER, and CIBERSORT-ABS) (**Figures 6C**, **E–I** **;** **Figures S6**-**9**). The proportions of tumor immune cells in different risk groups are graphically displayed in **Figure 6A**, and **Figure 6B** illustrates their distributions as boxplots. The group with low LNPRS had higher proportions of naive B cells, CD8^+^ T cells, and resting dendritic cells than the group with high LNPRS (P-value< 0.01; Wilcoxon test; **Figure 6B**). However, the proportions of M0 macrophages and resting NK cells in the high LNPRS group were significantly higher than those in the low LNPRS group (P-value< 0.05; Wilcoxon test; **Figure 6B**). **Figure 6C** suggests that the proportions of TILs, including CD8^+^ T cells, CD4^+^ T cells, and myeloid dendritic cells, were statistically inversely correlated with LNPRS by using at least 4 algorithms (P-value< 0.05; Wilcoxon test; **Figure 6C**, **E–I** **;** **Figures S6**-**9**). The proportions of regulatory T cells (Tregs) and M0 macrophages were positively correlated with LNPRS by using the CIBERSORT algorithm (P-value< 0.01; Wilcoxon test; **Figure 6C**) (20).

In addition, the relationship between five genes based on the LNPRS and 22 immune cell types was also evaluated in the training cohort by using 7 algorithms (**Figures 6J–Q** **;** **Figure S10**). The correlations between GBP2 expression and TILs, including macrophages, M1 macrophages, NK cells, resting memory CD4^+^ T cells, CD4^+^ T cells, CD8^+^ T cells, and myeloid dendritic cell-activated neutrophils, were significantly positive (**Figures 6J–Q**). The relationship between the expression of GBP2 and SLAIN1 and TILs was also assessed by using different algorithms (**Figure S10**).

### Correlation of LNPRS, immune checkpoint molecules, and antitumor immunotherapy response

**Figure 7:**
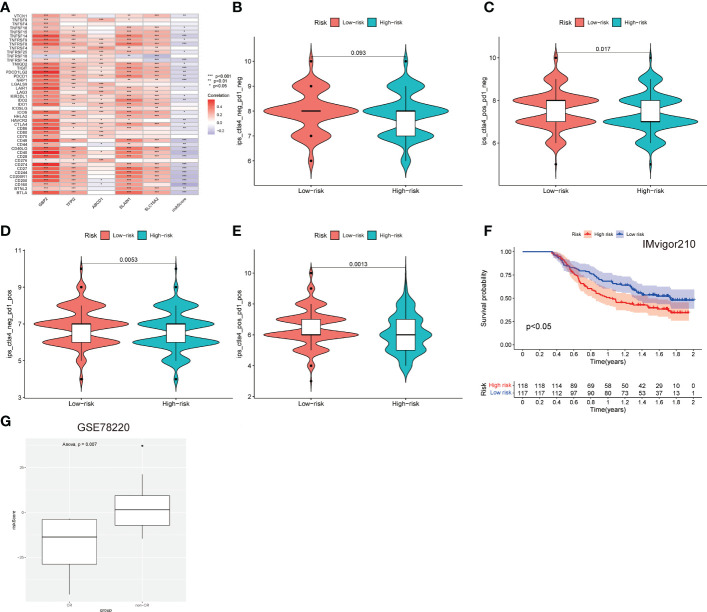
The predictive value of the immunotherapy efficacy of the LNPRS in the 3 independent cohorts. **(A)** The correlation between immune checkpoints, gene expression, and the LNPRS. **(B–E)** The IPS and response to blockade with CTLA-4 antibody or/and PD-1 antibody for the TCGA cohort. **(F)** The survival curves for the Imvigor210 cohort. **(G)** In the GSE78220 cohort, the differences in the LNPRS of patients with distinct chemotherapy responses.

The associations of 45 important immune checkpoint molecules and the LNPRS for the TCGA cohort, including IDO1, LAG3, and CTLA-4, were investigated (**Figure 7A**). The expression of 16 immune checkpoint molecules was inversely correlated with the LNPRS, including BTLA, CD244, CD27, CD274, CD40, CD48, NRP1, TIGIT, TNFRSF8, CD28, CD160, CD200, CD200R1, TNFRSF14, and CD40LG (**Figure 7A**). Whether treated with PD-1 antibody alone or in combination with CTLA-4 antibody, patients with low LNPRS received higher IPS scores than those with high LNPRS (**Figures 7B–E**).

Then, we assessed the predictive value of the efficacy and survival time of the LNPRS in the Imvigor210 cohort and GSE78220 cohort with PD-L1 antibody or PD-1 antibody (**Figures 7F, G**). The patients of the two cohorts were divided into high or low LNPRS groups by the same formula. In the Imvigor210 cohort, the patients with low LNPRS had longer overall survival than those with high LNPRS (1.02 years versus 1.74 years, P-value = 2.1E-2; log-rank test; **Figure 7F**). According to the response of patients to immunotherapy in the GSE78220 cohort, the breast cancer patients were sorted into four groups, and the responses to immunotherapy were progressive disease (PD), partial response (PR), stable disease (SD), and complete response (CR). A noticeable difference in LNPRS with different responses to chemotherapy was observed in the GSE78220 cohort and the patients in the non-CR groups had higher LNPRS than the patients with a complete response (P-value = 7.0E-3; t-test; **Figure 7G**).

### The predictive value of LNPRS for the responses to chemotherapy

**Figure 8:**
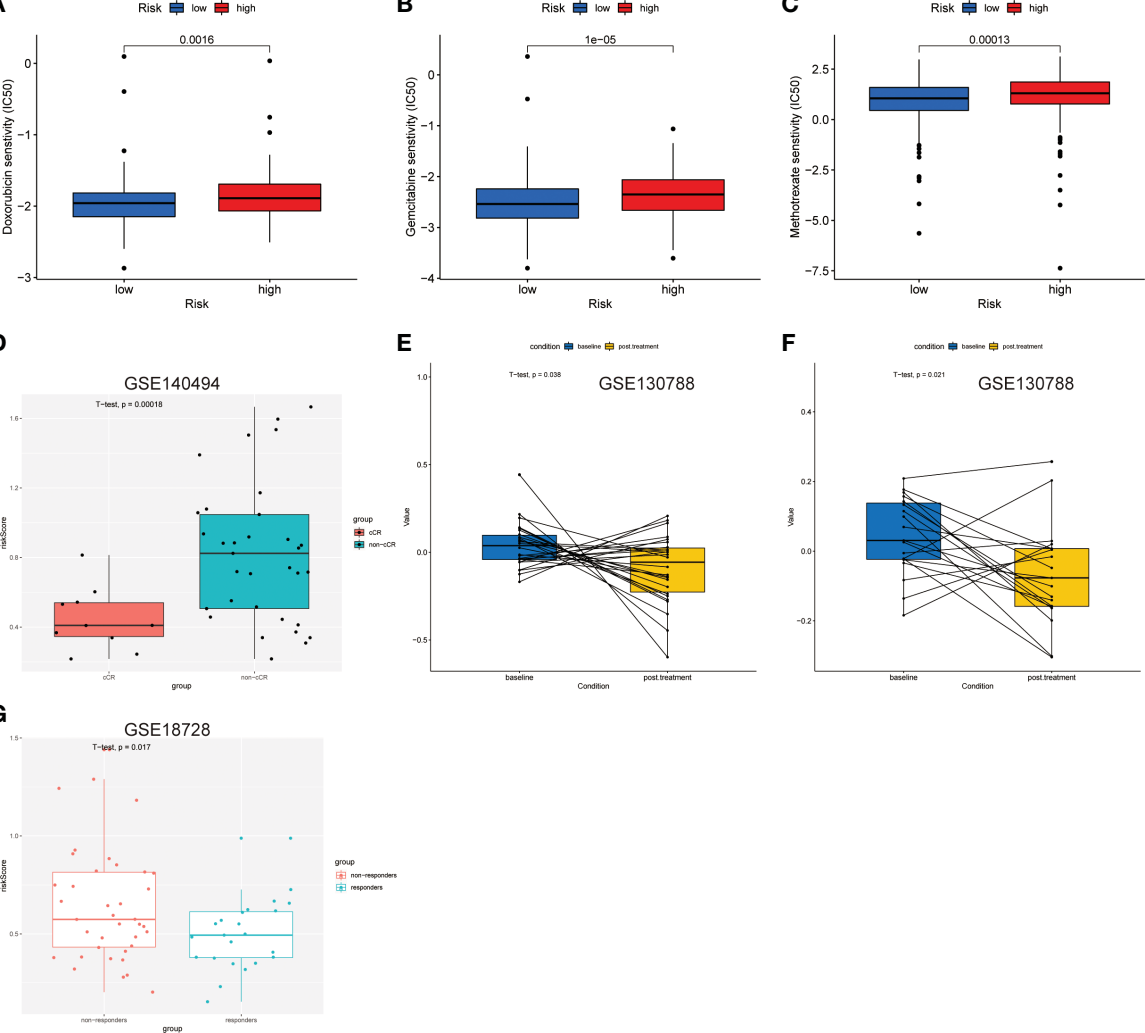
Predictive value of the LNPRS for chemotherapeutic efficacy in the 4 independent cohorts. **(A–C)** In the TCGA cohort, the differences in sensitivity to chemotherapy drugs of patients with low and high LNPRS. **(D, G)** The boxplot shows the difference in the LNPRS for breast cancer patients with different responses to neoadjuvant chemotherapy in the GSE140494 **(D)** and GSE18728 **(G)** cohorts. Significance was determined by a t-test. **(E, F)** Pairwise comparison of the LNPRS in breast cancer patients pre- and postchemotherapy for the GSE130788 cohort by the pairwise t-test.

The sensitivity differences of the 138 cytotoxic drugs, including doxorubicin, gemcitabine, and methotrexate, were studied in the groups with high and low LNPRS (**Figures 8A–C** **;** **Figure S11**). The sensitivity differences of the 23 drugs were identified between different groups, and the P-values for doxorubicin, gemcitabine, and methotrexate were 1.6E-3, 1.0E-5, and 1.3E-4, respectively (**Figures 8A–C** **;** **Figure S11**).

Next, we assessed the predictive value of LNPRS in the GSE140494, GSE130788, and GSE18728 cohorts (**Figures 8D–G**). The patients in the GSE140494 cohort were sorted into three groups based on the response to neoadjuvant chemotherapy: clinical no change (cNC), clinical partial response (cPR), and clinical complete response (cCR). The noticeable difference in LNPRS between the cCR and non-cCR groups was investigated in the GSE140494 cohort at the time point of 6 cycles, and the patients with non-cCR had higher LNPRS than those with cCR (P-value = 1.8E-4; t-test; **Figure 8D**).

Then, we further verified the predictive value of LNPRS for neoadjuvant chemotherapy in the GSE18728 cohort and obtained similar results (**Figure 8G**). The breast cancer patients were sorted into responders and non-responders based on therapeutic efficacy. Compared to the LNPRS in the non-responder group, lower LNPRS were found in the responder group (P-value = 1.7E-2; t-test; **Figure 8G**).

Finally, the GSE130788 cohort was used as a validation cohort to verify the value of LNPRS by performing pairwise comparisons. In the GSE130788 dataset, breast cancer patients received neoadjuvant chemotherapy, and the treatment options included TCH, TCTy, and TCHTy. **Figures 8E, F** demonstrates that the breast cancer patients after TCH treatment had significantly lower LNPRS than the scores of patients before treatment (baseline) (P-value = 3.8E-2; pairwise t-test; **Figure 8E**), and the result was consistent in the group that received TCTHY treatment (P-value = 2.1E-2; pairwise t-test; **Figure 8F**).

### GSEA and GSVA analyses of LNPRS

**Figure 9:**
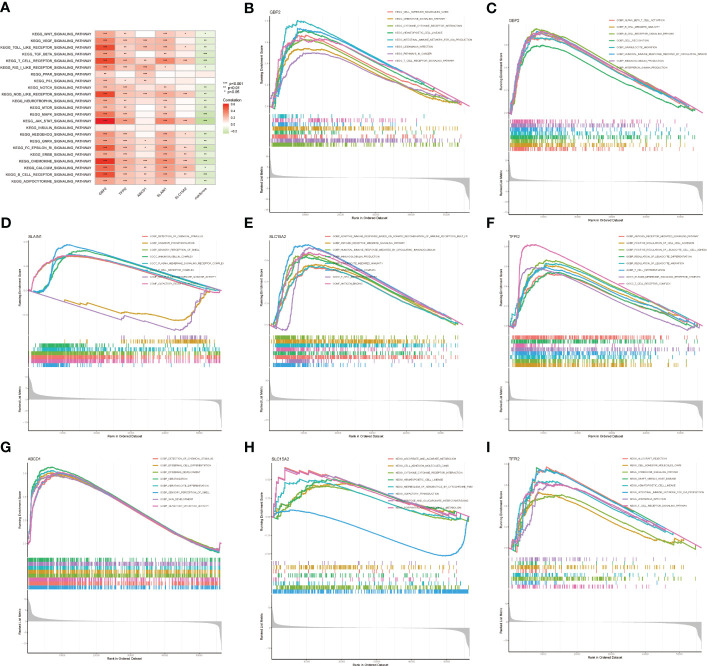
GSVA and GSEA enrichment analysis based on LNPRS in the TCGA cohort. **(A)** KEGG signaling pathways of genes that independently influence prognosis and LNPRS. **(B–I)** KEGG and GO enrichment analysis of five genes based on LNPRS.

GSVA enrichment analysis was performed to elucidate which signatures were significantly related to the LNPRS. In total, 7 KEGG pathways were identified, as illustrated in **Figure 9A**, and these KEGG pathways were negatively correlated with the LNPRS. The KEGG enrichment pathways included T_BETA_SIGNALING, T_CELL_RECEPTOR_SIGNALING, CHEMOKINE_SIGNALING, and B_CELL_RECEPTOR_SIGNALING, MAPK_SIGNALING, JAK_STAT_SIGNALING and FC_EPSILON_RI_SIGNALING (P-value< 1.0E-3; **Figure 9A**). GSEA included GO and KEGG pathways based on single genes (**Figures 9B–I**). The GO enrichment pathways for GBP2 included ALPHA_BETA_T_CELL_ACTIVATION, B_CELL_RECEPTOR_SIGNALING_PATHWAY, HUMORAL_IMMUNE_RESPONSE_MEDIATED_BY_CIRCULATING_IMMUNOLOGY, IMMUNOGLOBULIN_PRODUCTION and INTERFERON_GAMMA_PRODUCTION (**Figure 9C**). **Figure 9B** illustrates that KEGG enrichment pathways for GBP2 were related to immunomodulation, such as T_CELL_RECEPTOR_SIGNALING_PATHWAY, INTESTINAL_IMMUNE_NETWORK_FOR_IGA_PRODUCTION, CYTOKINE_RECEPTOR_INTERACTION, and CHEMOKINE_SIGNALING_PATHWAY. The GO and KEGG enrichment analyses for TFPI2, SLC15A2, SLAIN1, and ABCD1 also obtained similar results in the training cohort (**Figures 9D–I**). The results of the study showed that the LNPRS was closely related to the activation and regulation of T lymphocytes, B lymphocytes, and immune cytokines.

## Discussion

Breast cancer patients with local lymph node metastases are at a high risk of forming distant metastases (29). It is important to test prognostic biomarkers and select some patients who are most likely to develop lymph node metastases or distant metastases. Potential and new prognostic indicators might enable oncologists to formulate a treatment plan for individual patients.

Immune cell infiltration is intimately associated with prognosis prediction and chemotherapy response for breast cancer patients (30). By co-expression analysis of mRNA expression data and immune cell infiltration for breast cancer patients with positive lymph nodes in the training cohort, a gene panel and scoring system based on immune cell infiltration characteristics that independently affected prognosis were explored and identified. In the multivariable Cox analysis, the LNPRS was proven to be a prognostic scoring system independent of other essential clinical features.

We studied the predictive prognosis performance of the LNPRS in 4 independent breast cancer cohorts. The breast cancer patients with high LNPRS had notably shorter overall survival times than those with low LNPRS in the training (TCGA) cohort (7.98 years versus 20.42 years, P-value< 8.16E-11), GSE20685 cohort (NA versus 14.1 years, P-value = 0.006), METABRIC cohort (10.16 years versus 13.05 years, P-value< 4.17E-4) and GSE97324 cohort (3.70 years versus NA, P-value = 0.017) by the log-rank test. Based on these results, we concluded that the negative correlation between the LNPRS and overall survival for breast cancer cohorts was significant. To further evaluate the predictive performance of the LNPRS in immunotherapy cohorts, the Imvigor210 cohort was examined, and there were also significant differences between the two groups with high or low LNPRS (1.02 years versus 1.74 years, P-value = 0.021).

Currently, chemotherapy is a very important therapeutic strategy for breast cancer patients. Thus, we assessed the predictive value of the LNPRS in the field of chemotherapy. The correlation results between LNPRS and drug sensitivity suggested that breast cancer patients with low LNPRS may benefit from chemotherapy with doxorubicin (P-value = 1.6E-3), gemcitabine (P-value =1E-5), and methotrexate (P-value = 1.3E-4). Therefore, we retrieved the data of breast cancer patients that received neoadjuvant chemotherapy or maintenance chemotherapy containing anthracycline from public datasets. The GSE140494 dataset not only met the above requirements but also included the efficacy evaluation information. Consequently, we used the GSE140494 cohort as a validation dataset to certify the predictive value of the therapeutic effect for the LNPRS. Unfortunately, we did not query the eligible public dataset that contained gemcitabine chemotherapy. The findings also confirmed that breast cancer patients with low LNPRS were more sensitive to small molecule tyrosine kinase inhibitors, including axitinib (P-value =2.6E-6) and nilotinib (P-value =4.8E-5). The breast cancer patients in the GSE130788 cohort also received combination treatment, which included lapatinib, and lapatinib was also a small molecule tyrosine kinase inhibitor. Therefore, the change in the LNPRS before and after treatment was observed and estimated in the GSE130788 cohort. The results demonstrated that the LNPRS can be a meaningful marker for predicting the response to chemotherapy of patients with breast cancer.

Some clinical studies confirmed that some patients can benefit from immune checkpoint treatment, and there were still some patients who did not benefit from immunotherapy with checkpoint inhibitors (31–33). The screening of sensitive predictors for immunotherapy is important for clinical treatment. First, we investigated the relationship between the LNPRS and the response to immune checkpoint treatment and then confirmed the prognostic value of the LNPRS in two immunotherapy cohorts. We identified that the LNPRS was related to the overall survival of patients and immunotherapy efficacy in the IMvigor210 and GSE78220 cohorts, and the results may uncover that the LNPRS may serve as a biomarker to predict the response to immunotherapy for breast cancer patients.

In our study, the prognostic value of the LNPRS in 4 breast cancer cohorts was examined. The clinicopathological parameters in some cohorts were limited, and some patients had no information about molecular subtypes. Therefore, we built the nomogram using breast cancer data from the training cohort, and the nomogram included some important clinical features except for molecular subtypes, such as TNM stage, clinical stage, sex, and age.

Next, the correlations between the LNPRS and enrichment pathways, immune checkpoint molecules, and the proportion of infiltrating immune cells were estimated. Differences in the TMB, ESTIMATE score, immune score, and stromal score were also observed in breast cancer cohorts. Based on the obtained results, we may presume that the LNPRS is a good biomarker for predicting prognosis in breast cancer patients.

There were some shortcomings in our study. First, the breast cancer patients were used as the training and testing cohorts from different public datasets in our study and the heterogeneity of tumors may exist in our cohorts. Previous studies have found that there was a correlation between neoantigen intratumor or intrapatient heterogeneity of tumors and overall survival (34). Tumor heterogeneity can determine the effects of chemotherapy and immunotherapy (2). Second, the LNPRS was established based on the TIME of breast cancer, and we did not obtain a public database of breast cancers for immunotherapy. Thus, we have to replace breast cancer cohorts with urothelial cancer and malignant melanoma datasets. Third, this study is based on bioinformatics analysis and lacks experimental validation. In the further study, we will collect transcriptome and clinical data from breast cancer patients in our studies and further verify the predictive value of the LNPRS by clinical validation.

Finally, the LNPRS model was a meaningful tool for survival prediction and therapy instruction for breast cancer patients with lymph node positivity. During the clinical treatment of breast cancer, the LNPRS model may help to predict prognosis and stratify patients who may benefit from adjuvant chemotherapy and immunotherapy with checkpoint inhibitors.

## Data availability statement

The datasets presented in this study can be found in online repositories. The names of the repository/repositories and accession number(s) can be found in the article/**Supplementary Material**.

## Ethics statement

The data generated in this study were derived from the public database. The patients/participants provided their written informed consent to participate in this study.

## Author contributions

XH and PW designed and supervised the study. NL and CF have written the manuscript. YY, LZ, XL and QZ have analyzed the data. All authors contributed to the article and approved the submitted version.

## Supplementary material

Supplementary Figure 1 Construction of the LNPRS in the training cohort. **(A)** The LASSO regression model was constructed by 10-fold cross validation. Partial likelihood deviance was calculated and displayed graphically. **(B)** LASSO coefficient profiles of 6 selected genes in the 10-fold cross validation.

Supplementary Figure 2 Validation of the LNPRS in the GSE20685 cohort. **(A, B)** The relationship between LNPRS and survival time in patients with breast cancer. **(C)** Expression heatmap of the 5 genes in different risk groups. **(D)** The ROC curves of the LNPRS model for the GSE20685 cohort.

Supplementary Figure 3 The proportion of important clinical features for the TCGA cohort with high or low LNPRS. **(A–D)** The bar graphs illustrate the composition of clinical features.

Supplementary Figure 4 The survival curves of patients with different TMBs in the different cohorts. **(A)** The survival curve with high or low TMB in the METABRIC cohort. **(B)** The ROC curves of the LNPRS model for the IMvigor210 cohort. **(C)** The ROC curves of the LNPRS model for the METABRIC cohort.

Supplementary Figure 5 The distribution of clinical features and survival curves of patients in the GSE97324 cohort. **(A–C)** The composition of clinical features is illustrated by the bar graphs; **(D)** The ROC curves of the LNPRS model. **(E, F)** The difference in the LNPRS of breast cancer patients with different T stages and stages.

Supplementary Figure 6 Correlation analysis of infiltrative immune cells with the LNPRS by the CIBERSORT-ABS algorithm in the TCGA cohort. **(A–K)** The dotted line plots show the relationship between the immune cells and the LNPRS.

Supplementary Figure 7 The correlation analysis of infiltrative immune cells with LNPRS by EPIC, TIMER, and QUANTISEQ algorithm in the TCGA cohort. **(A–Q)** The dotted line plots show the relationship between the immune cells and the LNPRS.

Supplementary Figure 8 Correlation analysis of infiltrative immune cells with the LNPRS by the MCPCOUNTER algorithm in the TCGA cohort. **(A–K)** The dotted line plots show the relationship between the immune cells and the LNPRS.

Supplementary Figure 9 Correlation analysis of infiltrative immune cells with the LNPRS by the XCELL algorithm in the TCGA cohort. **(A–Q)** The dotted line plots show the relationship between the immune cells and the LNPRS.

Supplementary Figure 10 The correlation analysis of infiltrative immune cells with LNPRS by 5 algorithms in the TCGA cohort. **(A–Q)** The relationship between immune cells and gene expression.

Supplementary Figure 11 The predictive value of the LNPRS for chemotherapy efficacy in the TCGA cohort. **(A–Y)** Differences in sensitivity to various chemotherapy drugs in the low and high-risk groups.
