# Supplementary material for: Immune microenvironment and clinical feature analyses based on a prognostic model in lymph node-positive breast cancer
Source: Front Oncol. 2023 Mar 22;13:1029070. doi: 10.3389/fonc.2023.1029070 (PMC10073659; doi:10.3389/fonc.2023.1029070)
Supplement: Supplementary file 1 [file Table_1.docx]

**Supplementary Table S1:** The correlation coefficients of different genes.

| **Gene** | **correlation coefficients** |
| --- | --- |
| GBP2 | -0.324261916142313 |
| TFPI2 | -0.211874749288829 |
| ABCD1 | 0.408631853994554 |
| SLAIN1 | -0.298900078746371 |
| SLC15A2 | 0.483996702845899 |
