# Supplementary figures and images for: Immune microenvironment and clinical feature analyses based on a prognostic model in lymph node-positive breast cancer
Source: Front Oncol. 2023 Mar 22;13:1029070. doi: 10.3389/fonc.2023.1029070 (PMC10073659; doi:10.3389/fonc.2023.1029070)

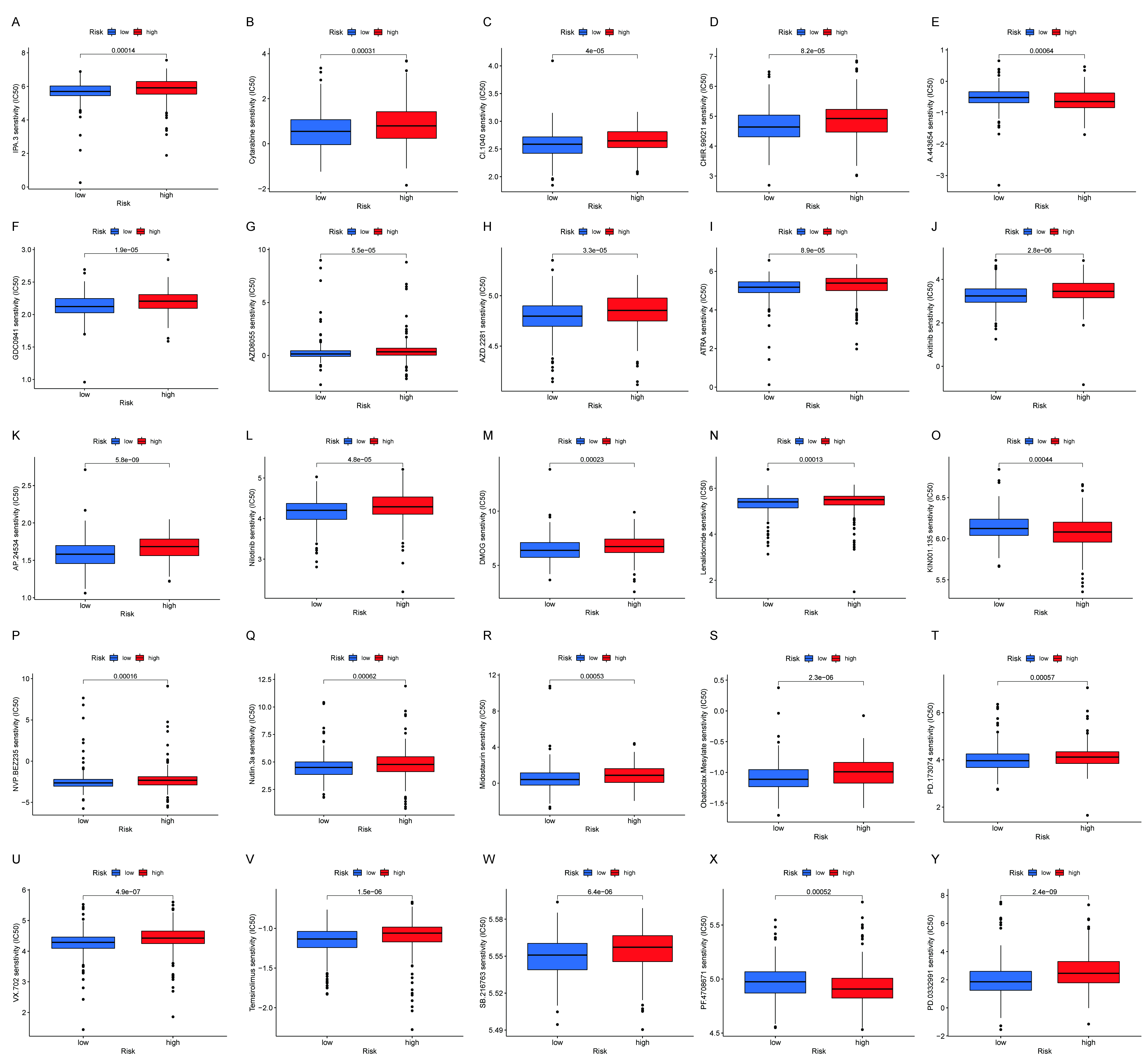

Supplement: Supplementary Figure 1 — Construction of the LNPRS in the training cohort. (A) The LASSO regression model was constructed by 10-fold cross validation. Partial likelihood deviance was calculated and displayed graphically. (B) LASSO coefficient profiles of 6 selected genes in the 10-fold cross validation. [file Image_1.tif]

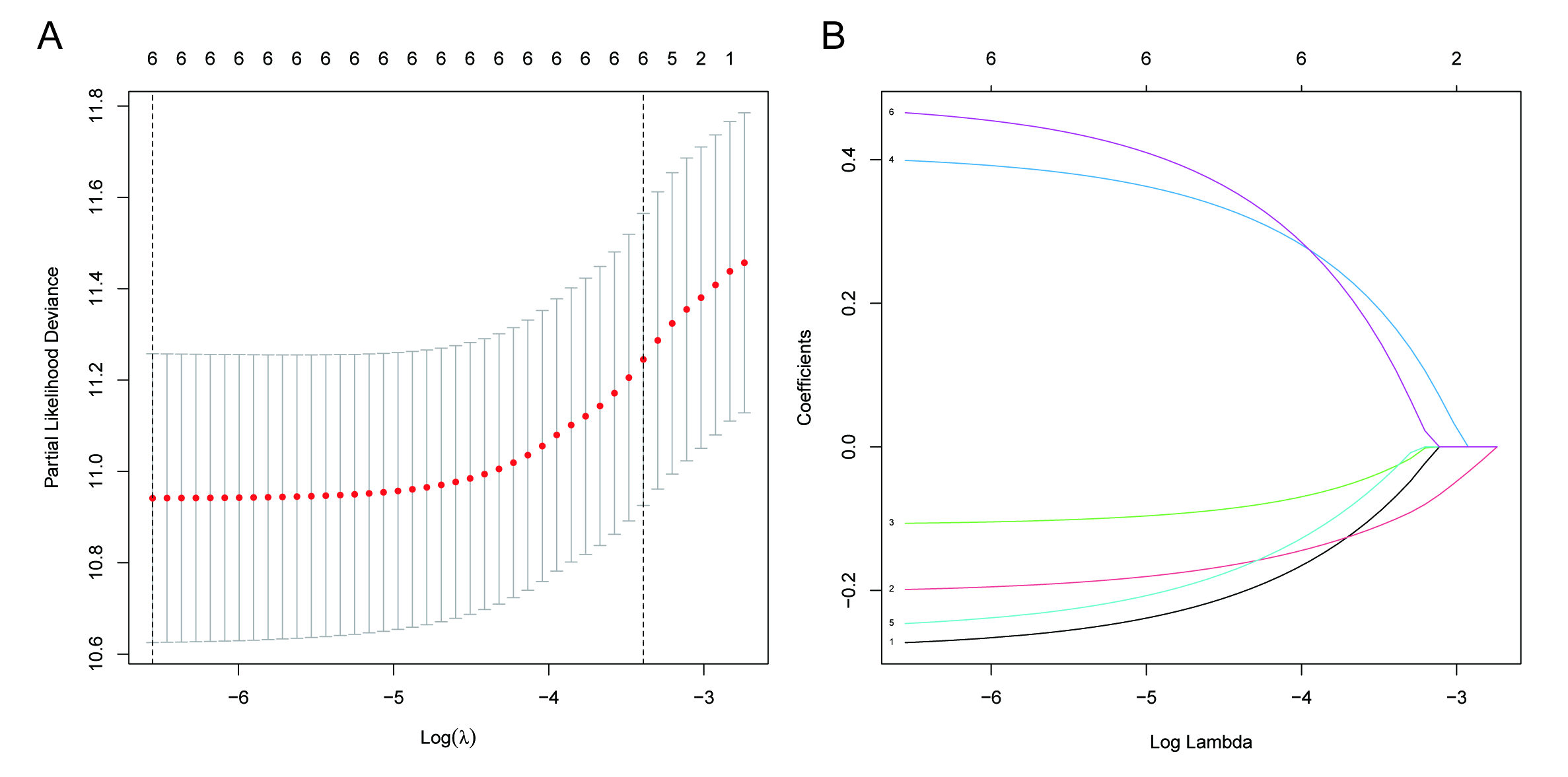

Supplement: Supplementary Figure 2 — Validation of the LNPRS in the GSE20685 cohort. (A, B) The relationship between LNPRS and survival time in patients with breast cancer. (C) Expression heatmap of the 5 genes in different risk groups. (D) The ROC curves of the LNPRS model for the GSE20685 cohort. [file Image_2.tif]

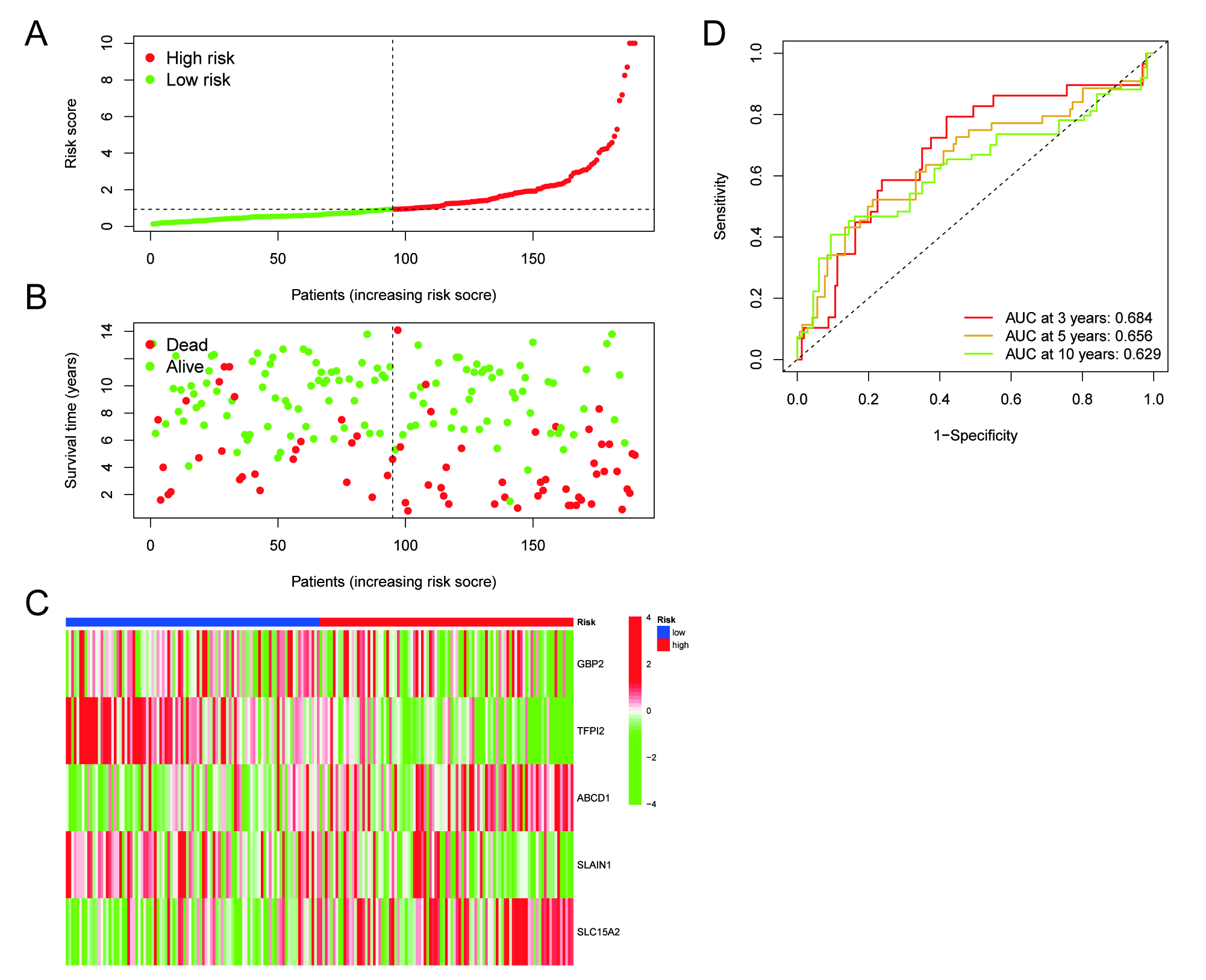

Supplement: Supplementary Figure 3 — The proportion of important clinical features for the TCGA cohort with high or low LNPRS. (A–D) The bar graphs illustrate the composition of clinical features. [file Image_3.tif]

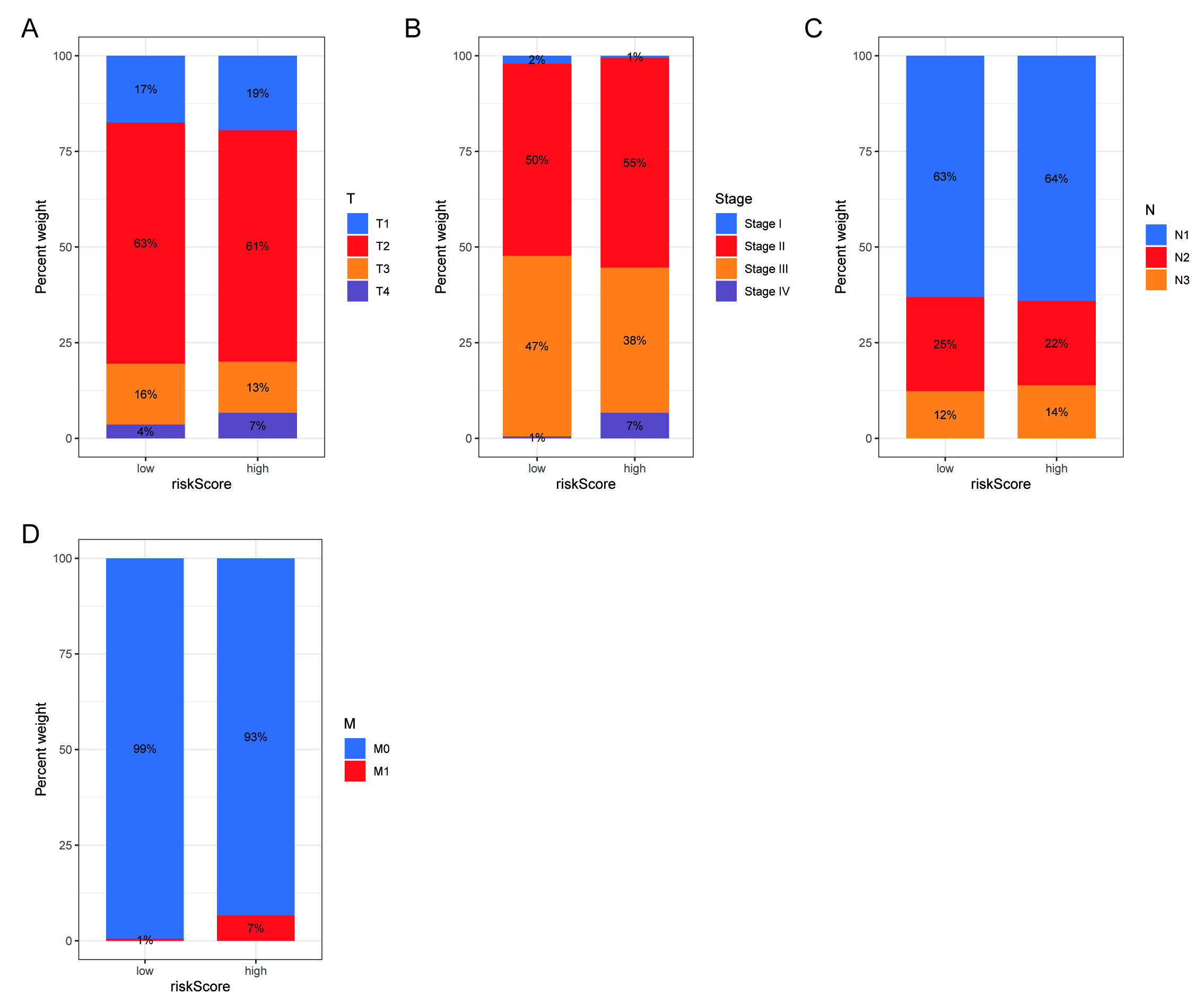

Supplement: Supplementary Figure 4 — The survival curves of patients with different TMBs in the different cohorts. (A) The survival curve with high or low TMB in the METABRIC cohort. (B) The ROC curves of the LNPRS model for the IMvigor210 cohort. (C) The ROC curves of the LNPRS model for the METABRIC cohort. [file Image_4.tif]

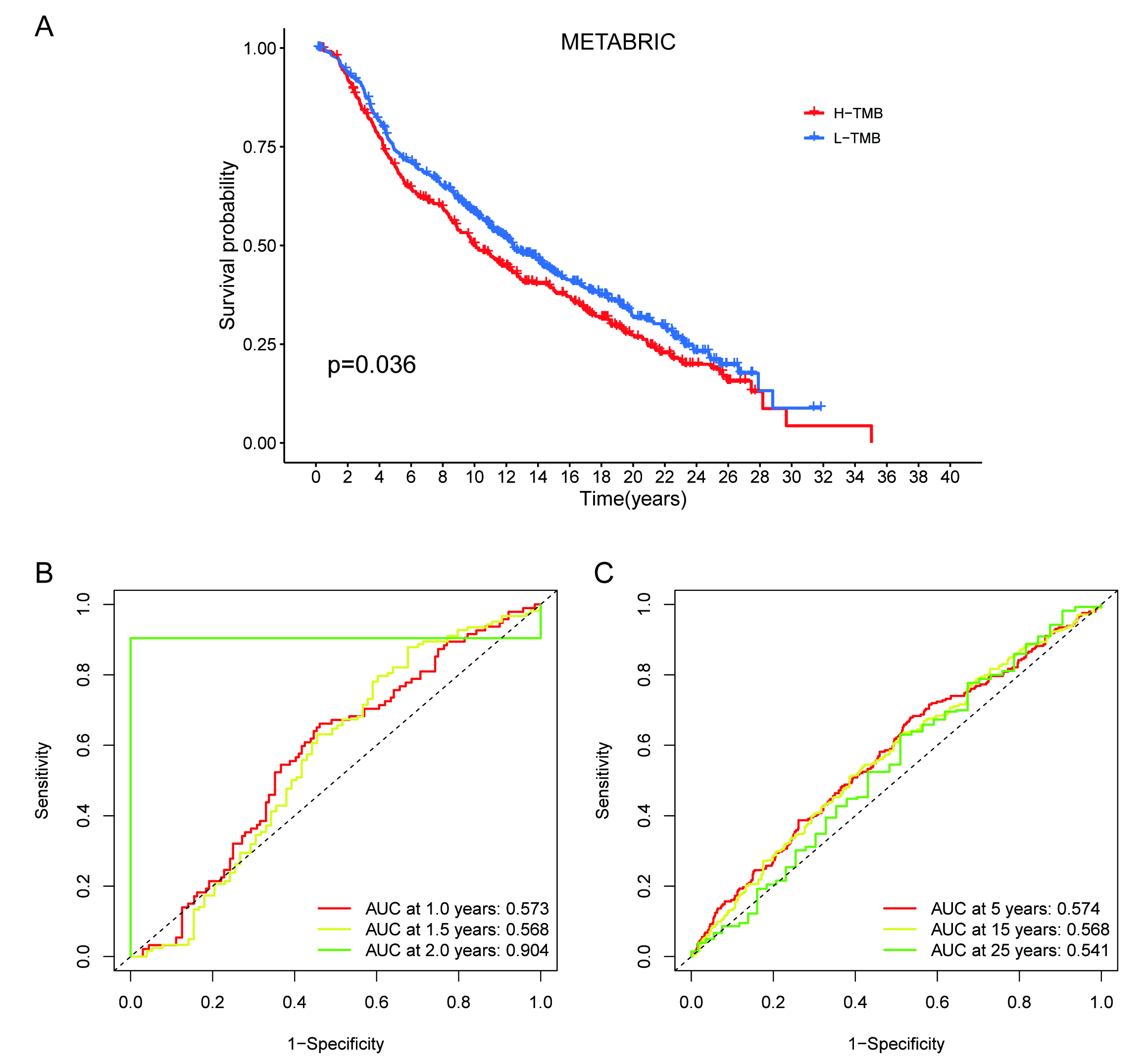

Supplement: Supplementary Figure 5 — The distribution of clinical features and survival curves of patients in the GSE97324 cohort. (A–C) The composition of clinical features is illustrated by the bar graphs; (D) The ROC curves of the LNPRS model. (E, F) The difference in the LNPRS of breast cancer patients with different T stages and stages. [file Image_5.tif]

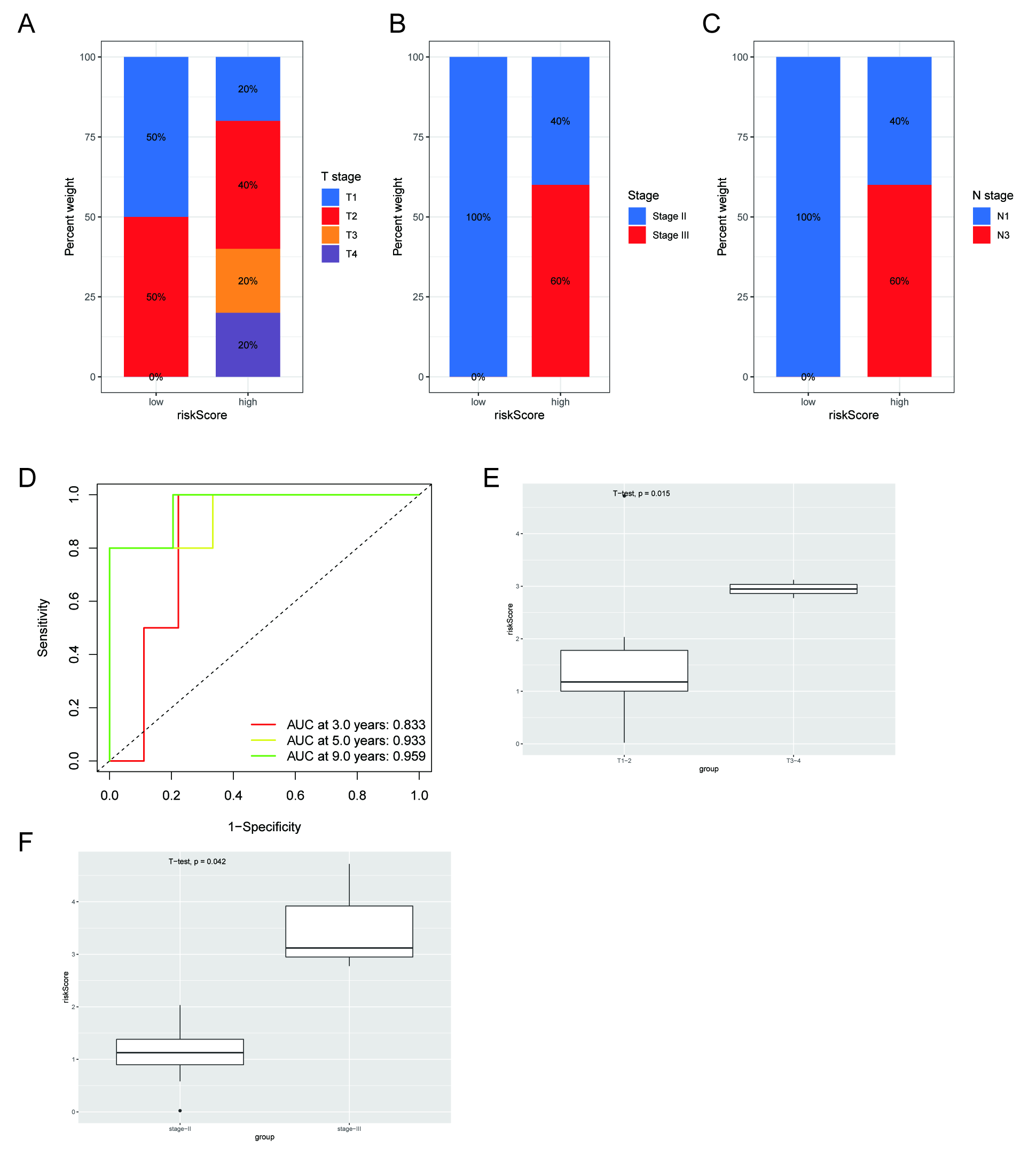

Supplement: Supplementary Figure 6 — Correlation analysis of infiltrative immune cells with the LNPRS by the CIBERSORT-ABS algorithm in the TCGA cohort. (A–K) The dotted line plots show the relationship between the immune cells and the LNPRS. [file Image_6.tif]

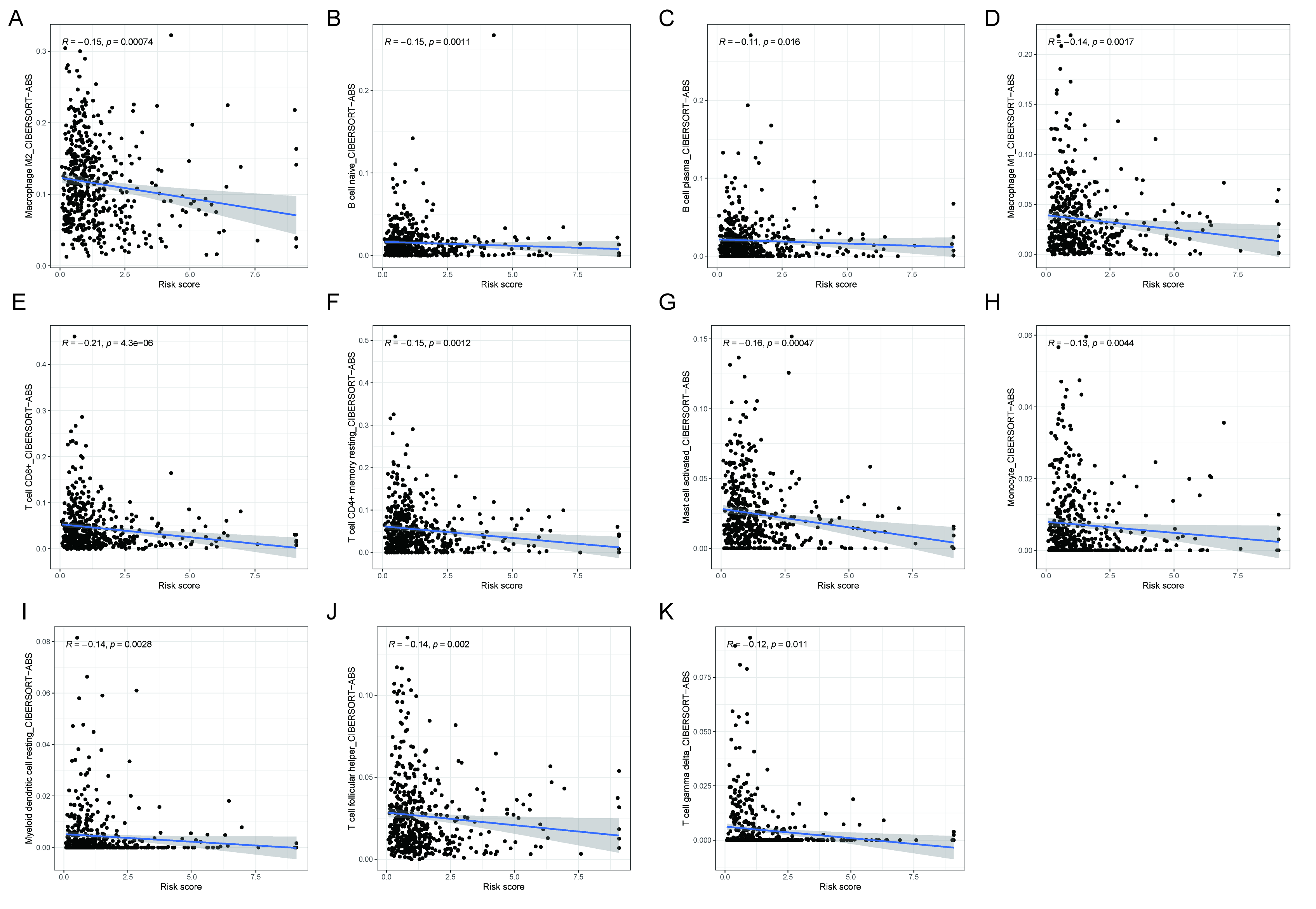

Supplement: Supplementary Figure 7 — The correlation analysis of infiltrative immune cells with LNPRS by EPIC, TIMER, and QUANTISEQ algorithm in the TCGA cohort. (A–Q) The dotted line plots show the relationship between the immune cells and the LNPRS. [file Image_7.tif]

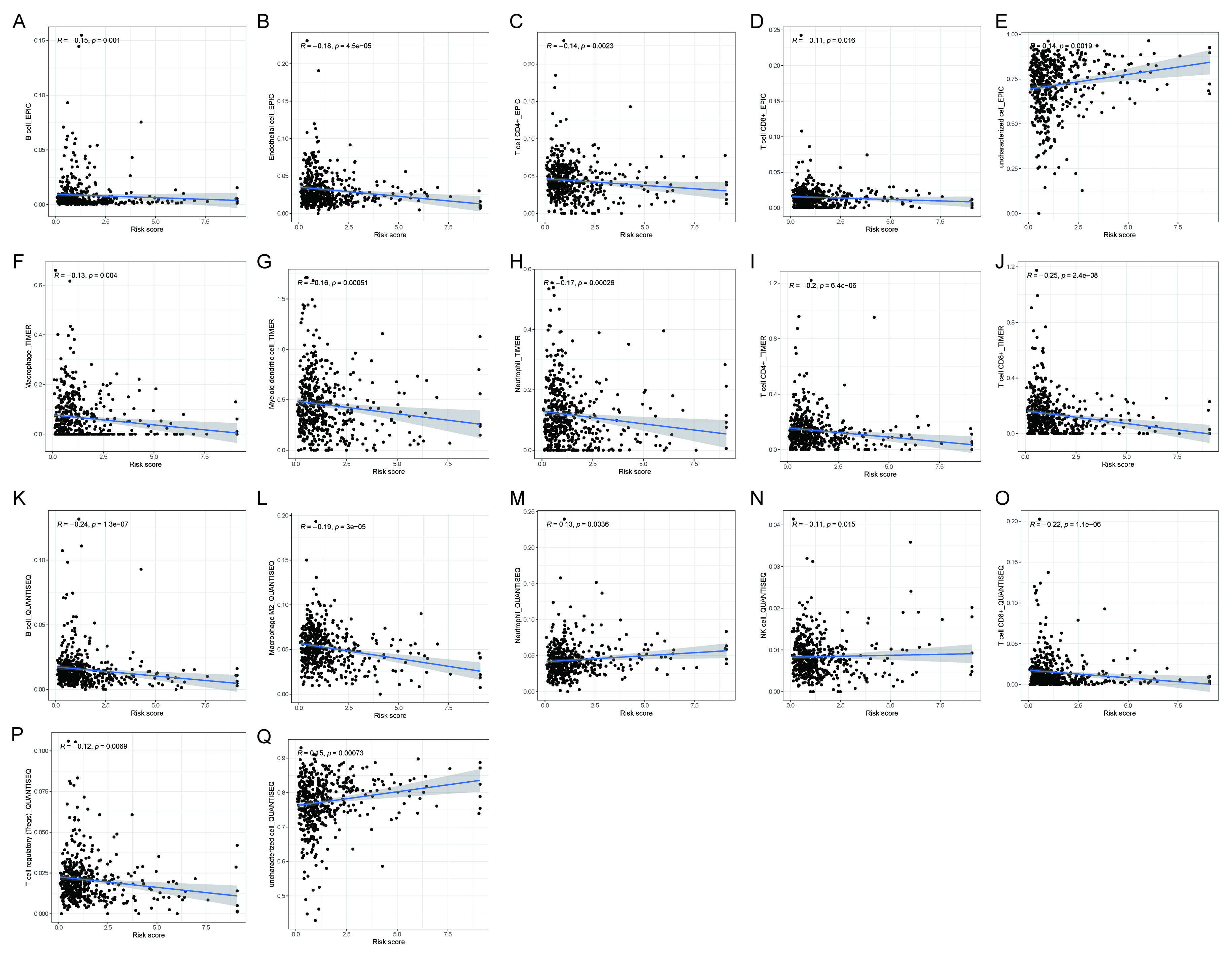

Supplement: Supplementary Figure 8 — Correlation analysis of infiltrative immune cells with the LNPRS by the MCPCOUNTER algorithm in the TCGA cohort. (A–K) The dotted line plots show the relationship between the immune cells and the LNPRS. [file Image_8.tif]

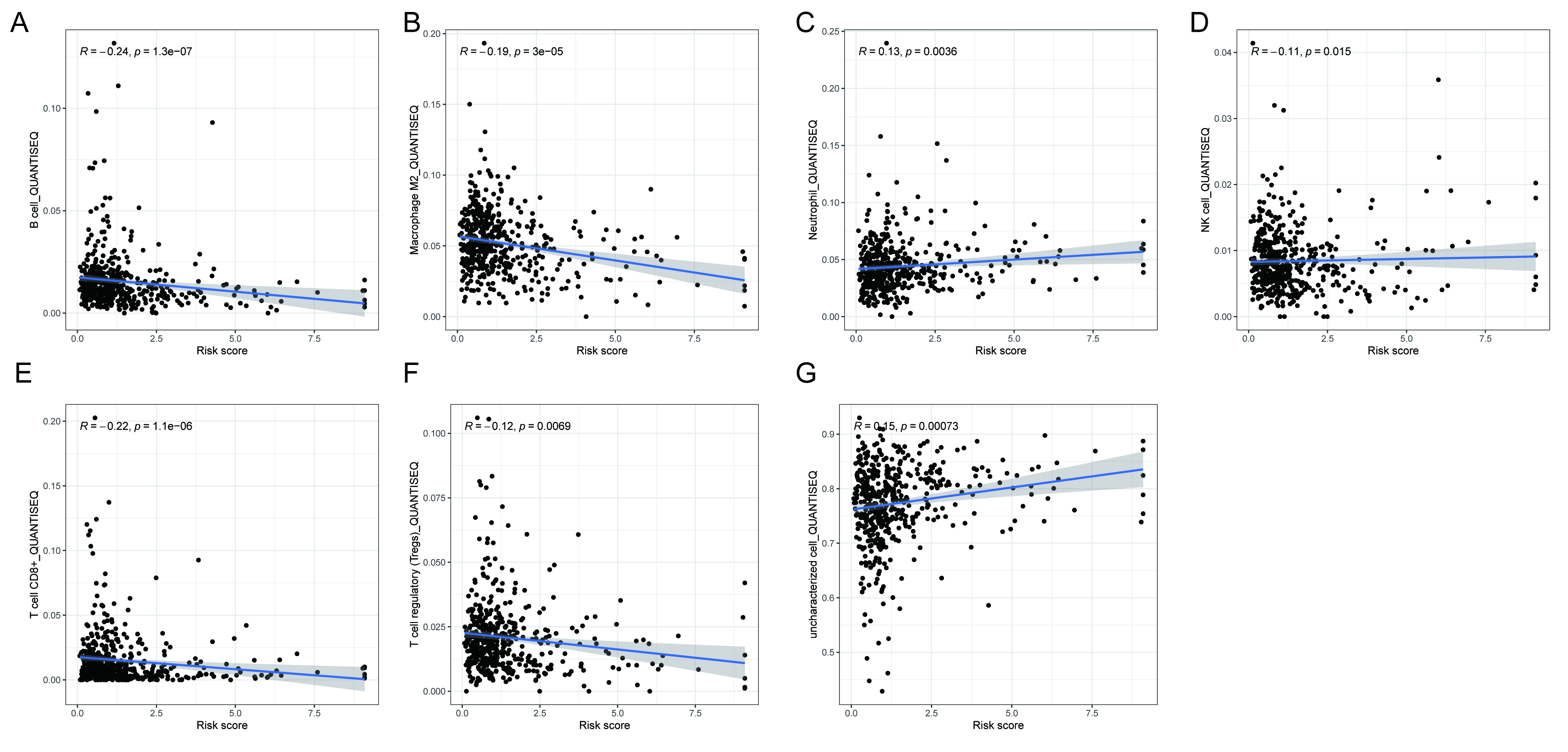

Supplement: Supplementary Figure 9 — Correlation analysis of infiltrative immune cells with the LNPRS by the XCELL algorithm in the TCGA cohort. (A–Q) The dotted line plots show the relationship between the immune cells and the LNPRS. [file Image_9.tif]

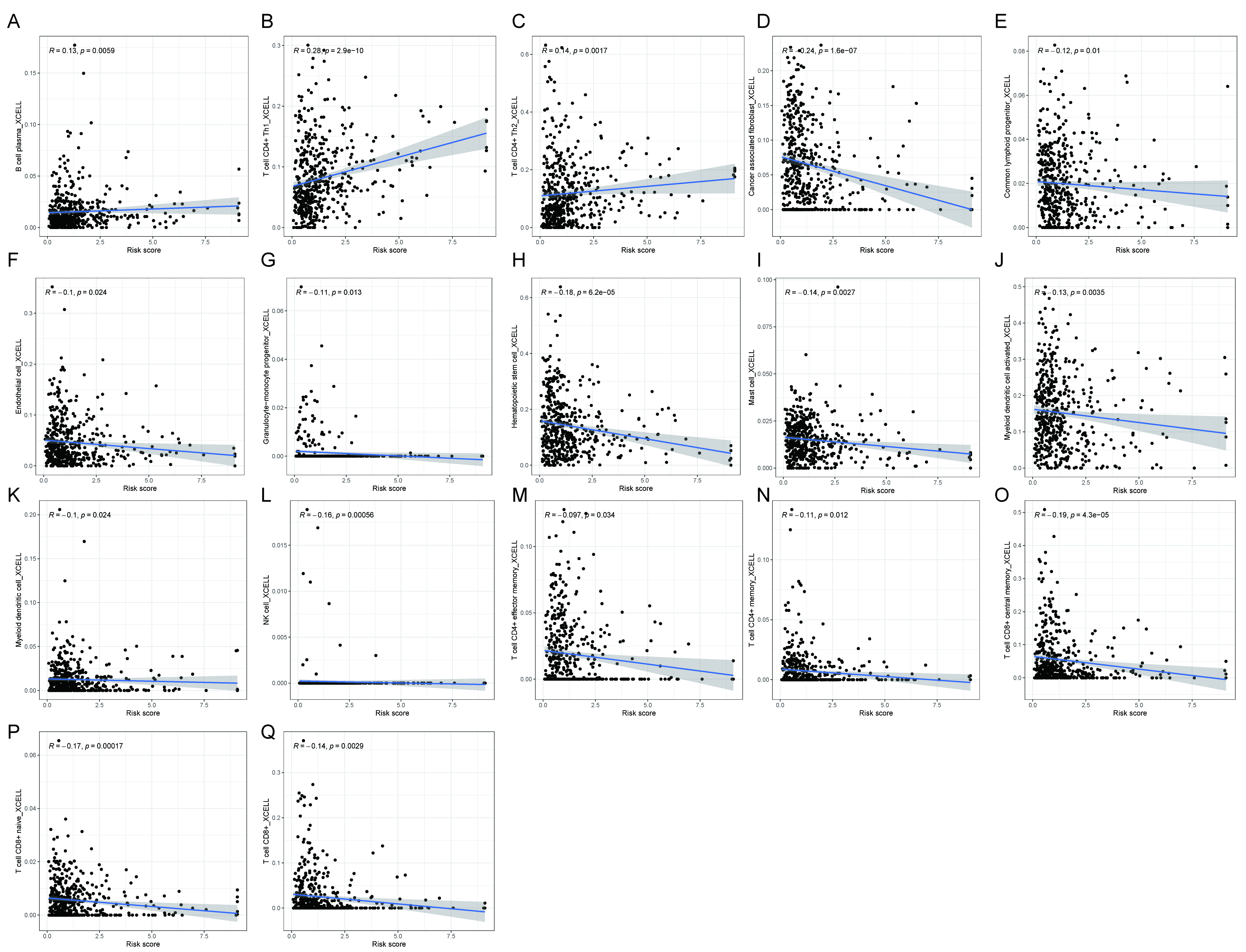

Supplement: Supplementary Figure 10 — The correlation analysis of infiltrative immune cells with LNPRS by 5 algorithms in the TCGA cohort. (A–Q) The relationship between immune cells and gene expression. [file Image_10.tif]

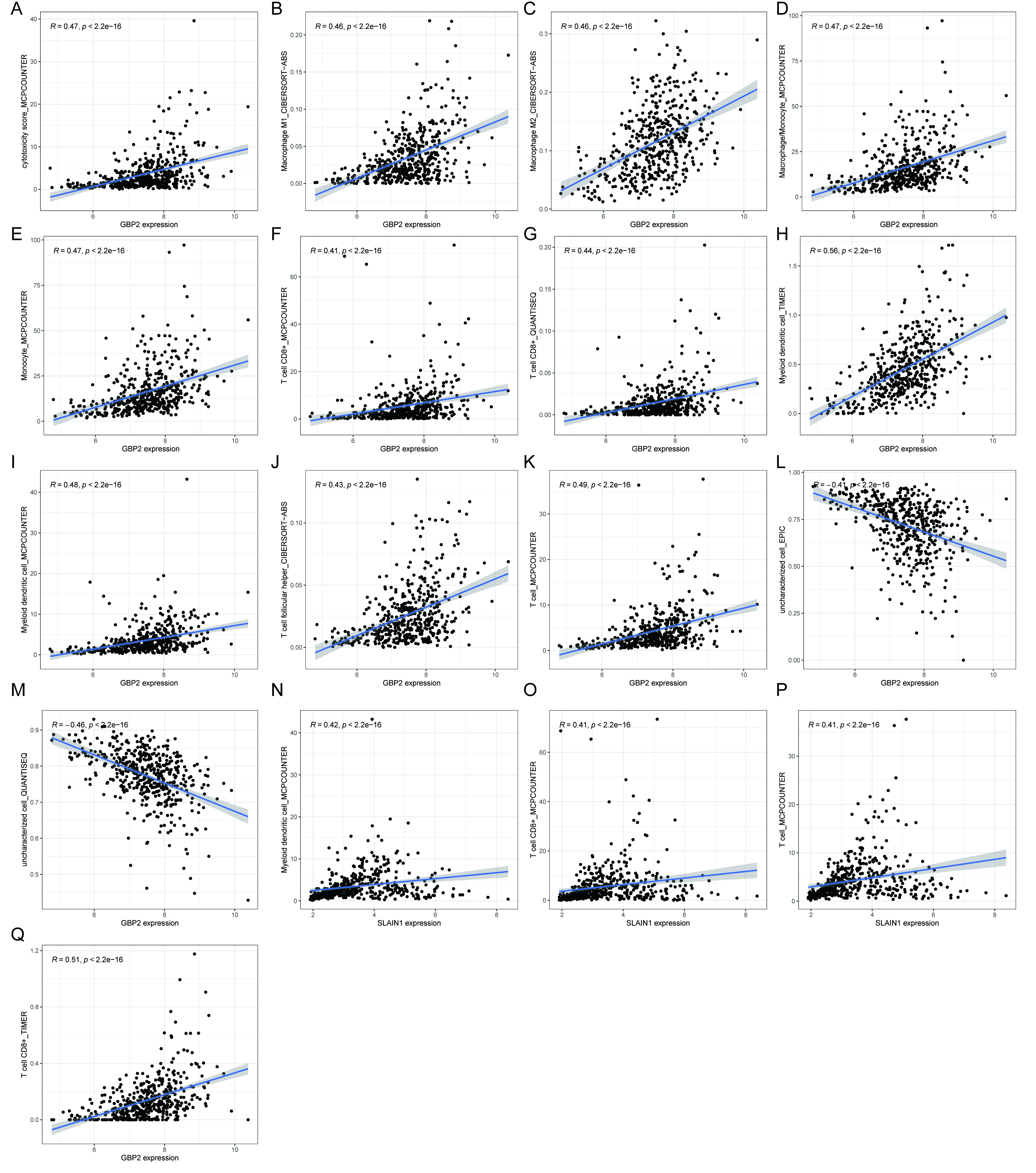

Supplement: Supplementary Figure 11 — The predictive value of the LNPRS for chemotherapy efficacy in the TCGA cohort. (A–Y) Differences in sensitivity to various chemotherapy drugs in the low and high-risk groups. [file Image_11.tif]
